# The Influence of Caregiver Burden and Mood Symptoms on Informant‐Reported Functional Abilities in Community‐Dwelling Older Adults

**DOI:** 10.1002/alz70857_101690

**Published:** 2025-12-25

**Authors:** Stephanie M. Simone, Moira McKniff, Marina Kaplan, Kimberly Halberstadter, Molly B. Tassoni, Sophia L. Holmqvist, Tania Giovannetti

**Affiliations:** ^1^ Temple University, Philadelphia, PA, USA

## Abstract

**Background:**

Informant reports are widely used to assess everyday functioning for the differential diagnosis of mild cognitive impairment (MCI) from dementia, to inform prognosis, and as outcomes in clinical trials. Functional ratings may be influenced by informant factors (e.g., education, relationship/role); however, little is known regarding the influence of informant depression/anxiety symptoms and caregiver burden on informant reports. This study investigated whether informant mood, anxiety, and distress influenced informant ratings of everyday functioning above and beyond participant demographics and cognitive abilities.

**Method:**

183 community‐dwelling older adults (*M* age = 73.57; 69% healthy cognition) completed questionnaires (demographics; Geriatric Depression Scale, 15‐item) and cognitive tests (global composite). Informants (*M* age = 63.75) completed demographic questionnaires, Beck Depression Inventory – Second Edition, State‐Trait Anxiety Inventory, Zarit Caregiver Burden Assessment, Functional Assessment Questionnaire (FAQ). Bivariate Spearman's rank‐order correlations or Kruskal‐Wallis tests were used to examine associations between participant and informant variables and FAQ. Hierarchical linear regression was used to explore the influence of informant depression/anxiety symptoms and caregiver burden on FAQ, with participant variables entered in Block 1 and informant variables added in Block 2.

**Result:**

Greater participant depressive symptoms (*r* = .265, *p* < .001) and worse cognition (*r* = ‐.350, *p* < .001) were significantly associated with higher (more impaired) FAQ ratings. Greater informant depressive symptoms (*r* = .177, *p* = .020), anxiety (*r* = .179, *p* = .020), and caregiver burden (*r* = .606, *p* < .001) were significantly associated with higher FAQ ratings. Participant and informant demographics were not associated with FAQ ratings (*p*s > .05). Regression results of Block 1 showed that only worse cognition was significantly associated with worse informant‐reported functioning (*B* = ‐.309, *p* < .001; Overall model: *R* ^2^ = 0.127, *F*(2, 159)=11.592, *p* < .001). Adding informant variables to Block 2 significantly improved the prediction model (R^2^ change = .242). In Block 2, worse cognition (*B* = ‐.136, *p* = .047) and greater caregiver burden (*B* = .521, *p* < .001) were significantly associated with worse informant‐reported functioning (Overall model: *R* ^2^ = 0.369, *F*(5, 156)=18.243, *p* < .001).

**Conclusion:**

A relation between high caregiver burden and over‐report of functional impairment has been shown in dementia samples. Our results show a similar association in a community‐based sample of mostly older adults without cognitive impairment. Researchers and clinicians should consider the influence of informant distress on their ratings of functional abilities.

